# Repetitive Daily Point of Choice Prompts and Occupational Sit-Stand Transfers, Concentration and Neuromuscular Performance in Office Workers: An RCT

**DOI:** 10.3390/ijerph120404340

**Published:** 2015-04-20

**Authors:** Lars Donath, Oliver Faude, Yannick Schefer, Ralf Roth, Lukas Zahner

**Affiliations:** Department of Sport, Exercise and Health, University of Basel, 4052 Basel, Switzerland; E-Mails: oliver.faude@unibas.ch (O.F.); yannick.schefer@stud.unibas.ch (Y.S.); ralf.roth@unibas.ch (R.R.); lukas.zahner@unibas.ch (L.Z.)

**Keywords:** standing time, sitting, sedentary behavior, point of choice prompts, intervention, balance, strength, worksite, stand desk, height-adjustable

## Abstract

*Objective:* Prolonged office sitting time adversely affects neuromuscular and cardiovascular health parameters. As a consequence, the present study investigated the effects of prompting the use of height-adjustable working desk (HAWD) on occupational sitting and standing time, neuromuscular outcomes and concentration in office workers. *Methods:* A single-blinded randomized controlled trial (RCT) with parallel group design was conducted. Thirty-eight office workers were supplied with HAWDs and randomly assigned (Strata: physical activity (PA), BMI, gender, workload) to a prompt (INT) or non-prompt (CON) group. INT received three daily screen-based prompts within 12 weeks. CON was only instructed once concerning the benefits of using HAWDs prior to the start of the study. Sitting and standing times were objectively assessed as primary outcomes for one entire working week using the ActiGraph wGT3X-BT at baseline (pre), after 6 (mid) and 12 weeks (post). Concentration (d2-test), postural sway during upright stance (under single, dual and triple task) and lower limb strength endurance (heel-rise) were collected as secondary outcomes. *Results:* With large but not statistically significant within group effects from pre to post, INT increased weekly standing time at work by 9% (*p* = 0.22, d = 0.8) representing an increase from 7.2 h (4.8) to 9.7 (6.6) h (*p* = 0.07). Concentration and neuromuscular performance did not change from pre to post testing (0.23 < *p* < 0.95; 0.001 < η_p_² < 0.05). *Conclusion:* Low-frequent and low cost screen-based point of choice prompts (3 per day within 12 weeks) already result in notable increases of occupational standing time of approx. daily 30 min. These stimuli, however, did not relevantly affect neuromuscular outcomes.

## 1. Introduction

Changes from physical to mainly cognitive work requirements within the last decades led to increased occupational sitting times, which adversely affect various health outcomes [1]. Sedentary behavior with energy expenditures of 1.5 or less metabolic equivalents (MET, multiples of the resting energy expenditure) [2] was reported to account for 6% of all-cause mortality and is in part independently linked to obesity, metabolic syndrome, type 2 diabetes, cardiovascular disease and musculoskeletal discomfort [3,4]. Mean daily sitting time being awake has been estimated as approx. 10 h [5,6]. The proportion of sitting time at the office was reported to range between 66 and 82% [7,8,9,10]. This variability could be attributed to differences in workplace culture and environments, types of work and might be due to different methods used to assess sedentary time. 

Several intervention studies that employed height-adjustable work desks (HAWD) revealed increases of occupational standing time ranging between 6 to 70 min (mean 30 min) per work day [11,12,13,14]. Methodological heterogeneity such as data collection (objective *vs.* questionnaire data acquisition), sample size, study duration, multi-componency of the interventional approach and participants’ baseline characteristics have been reported to mainly account for this variability. Only one study that enrolled public health researchers who were aware of the topic and its relevance achieved sitting time reductions of about 2 h per 8-h work day [15]. Daily office sitting time reductions of between 60 to 90 min are more likely achievable when employing more complex and expensive multi-component programs operating on organizational (consulting, education, scheduling) and individual (coaching, phone, mails) levels [16]. 

Feasible and cost-efficient point of choice prompts (e.g., reminding poster, pop-up windows) have been successfully applied to promote health-enhancing behavior (e.g., stair climbing instead of elevator use) [17]. Based on this idea, the provision of high-frequent (e.g., every 30, 45 or 60 min) screen-based point of choice prompts on physical activity promotion (walking or standing up) were beneficially applied to increase daily energy expenditures (+190 kcal per day in a prompting situation) and standing time (+30 min/day) at work [7,18,19]. As high-frequently appearing screen-based prompts could be increasingly ignored over time and might bother employees when appearing too often at inappropriate moments, organizations should reduce screen-based prompting to an effective minimum [19]. 

As prolonged standing has been shown to reveal higher caloric energy expenditures and cardiac responses compared to sitting [20], it seems interesting to assume that concentration capacity may also benefit from increased periods of daily occupational standing. Increased occupational standing might also result in better neuromuscular performance, since lower limb muscles (e.g., plantar flexor muscles) are greater activated during upright standing compared to sitting [21]. In this regard, one previous study revealed that only three daily eight minutes bouts of work-site balance and strength training (8 weeks in total) can remarkably improve postural control and force development in middle-aged office workers [22]. 

Against this background, the present study aimed at examining the effects of daily point of choice prompts on standing time increases in office workers. We hypothesized that already low-frequent and repetitive screen-based prompts (three times per day) can result in relevant increases of daily office standing time in healthy middle-aged office workers. We additionally considered that standing balance and strength performance as well as concentration as secondary outcomes might benefit from increased daily standing time. 

## 2. Methods 

### 2.1. Study Design and Participants

**Figure 1:**
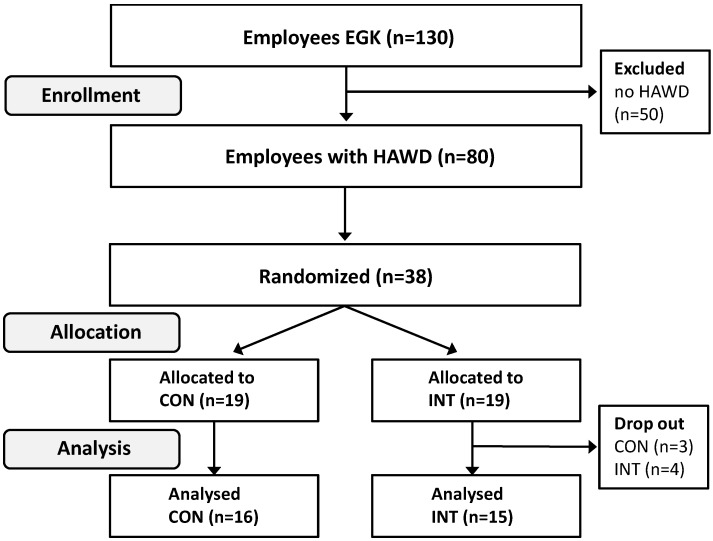
Participant flow throughout the study.

The present study was conducted as a single-blinded randomized controlled trial (RCT) with a twelve weeks lasting intervention period. The confederate Swiss health insurance (EGK, mainly females with mid-school education) with 130 employees was initially asked to participate in the study (Figure 1). All office workers who were supplied with a HAWD and did not use it yet (n = 80) were invited to participate in the study (Figure 1). Of those 80 employees, 42 did not meet the inclusion criteria or were not willing to participate in the study. Participants with cardiovascular disease, trauma of the lower extremities in the past six months, epilepsy, pregnancy and dizziness were not allowed to be included in the study. Few of those participants who were assigned to the groups dropped out due to job changes (n = 2) and illness (n = 5). Office workers of both sexes between 18–65 years of age, working at least part-time (minimum 50%) were enrolled in the study. All participants received information on the schedule of the study and the methods. Hypotheses were not communicated in order to meet blinding criteria. All subjects gave their informed consent for inclusion before they participated in the study. The study was conducted in accordance with the Declaration of Helsinki, and the protocol was approved by the Ethics Committee of the University of Basel (2013/141). Data were collected, stored and analyzed pseudonymized. Thirty-eight volunteers remained in the study and were randomly assigned to either the control group (CON) or the intervention group (INT) (Table 1). 

**Table 1:** Demographical data of the participants at before (pre) and after (post) the 12 week intervention for the intervention group (INT) and control group (CON). Data are presented as means ± standard deviations (SD).

Demographical data	INT (n = 15)				CON (n = 16)			
gender (m/f)	4/11				4/12			
	Pre		Post		Pre		Post	
age (years)	45 (12)				40 (10)			
height (cm)	167.4 (9.0)				168.7 (10)			
weight (kg)	65.8	(12.9)	65.4	(12.7)	70.4	(15.7)	69.9	(14.0)
BMI (kg/m²)	23.7	(3.7)	23.7	(3.7)	24.7	(5.0)	24.6	(4.3)
body fat (%)	28.2	(7.3)	27.4	(7.2)	28.3	(8.3)	29.0	(7.9)
physical activity (h/week)	6.2	(5.0)	7.0	(4.7)	6.2	(4.0)	7.1	(7.6)
working time (h/week)	39	(8)	39	(9)	36	(9)	36	(9)

Body fat was assessed via bio-impedance analyses using the Inbody [23]. Summed hours of general and sportive physical activity were assessed using the Freiburger physical activity questionnaire [24]. 

Group assignment was randomly conducted according to the minimization method: Thereby, age, gender, body mass index (BMI), physical activity and working time served as strata criteria in order to minimize group differences in demographical variables [25]. Testing personnel was blinded to group allocation. Sitting and standing times were collected via accelerometry for one respective working week at baseline, within the 6th and 12th intervention weeks. Secondary outcomes (postural sway during upright stance, strength endurance of the lower limb as well as concentration) were only examined at baseline and after completing the intervention period (after 12 weeks). Finally, 16 participants of CON and 15 participants of INT were analyzed (Figure 1). This sample size enables to detect small to moderate (η_p_² = 0.07) effects with a power of 90% and a significance level of 5%. 

### 2.2. Outcome Measures

#### 2.2.1. Standing and Sitting Time Assessment

Objective sitting and standing times were assessed using the ActiGraph wGT3X-BT (Pensacola, FL, USA). This small tri-axial accelerometer with integrated inclinometer function enables the detection of sitting, stepping and standing [26]. Although the ActiGraph monitor is usually worn on the hip, in this study the monitor was worn on the right thigh (over the trousers, comfortably attached with an elastic belt), using the inclinometer function to assess the time spent in sitting and standing position. The ActiGraph has been reported to serve as a valid instrument to distinguish between standing and sitting posture worn at the thigh, described in detail elsewhere [27]. Briefly, the ActiGraph worn at the thigh uses raw data to estimate time spent in three categories: sitting/lying, standing, and moving. “Moving” in this case is determined by considering movements not step counts [27]. Generally, this approach has been reported to provide high sensitivity (~99%) and specificity (100%) in distinguishing between sitting, standing, walking, running and cycling, described in detail elsewhere [28]. Briefly, the standard deviations of acceleration enable the classification between seven different classifications (sitting, standing, walking, running, stair walking, cycling and “move”) using a tree structure in order to classify activity levels. However, we did not differentiate seven classification patterns. Data were captured during one working week for five consecutive working days before the intervention period (baseline) and within the 6th and 12th interventional week. The participants were instructed to put on the ActiGraph when they started the working day. After finishing their working day they were allowed to remove the ActiGraph. The devices were initialized to record data at a sampling rate of 60 Hz for all three axes processed at 1 min epoch level [27]. Data were downloaded using the corresponding ActiLife 6 software. The information of each epoch length were summed up and further analyzed. Wear and non-wear time can be reliably detected using this procedure [29]. All five working days of each participant could have been analyzed validly as wear time has been also double checked by the manual wear time recordings and the stamping system (work time keeping) of the company. Thus, no cut points, for wear and non-wear time classification was mandatorily required. A too low cut-point also aggravates a valid detection of sitting times. This was important as participants were allowed to remove the ActiGraph during leisure time and overnight. We merely focused on collecting the time sitting and standing at work. Sitting and standing times were provided in absolute hours and percentages of total working time. Participants were also asked to manually record the times (hours and minutes) on a printed schedule when changes of body position (sitting, standing, walking) were done. Thereby, the exact time was noted. 

#### 2.2.2. Concentration

The test d2 of Brickenkamp (1962) is a paper and pencil test used to examine attention and concentration processes [30]. This test contains 14 rows, each with 47 interspersed characters. A time limit of 20 sec for each row was provided by the test instructor. Participants received a standardized instruction on the testing modalities and executed an example line of 22 characters according to the test manual. The participants were instructed to cross out as many target characters as possible within 20 sec (“d” with a total of two dashes either above or below) while ignoring non-target characters (“d” with more or less than two dashes, and any “*p*”). The participants performed the test at different times of the working day but within individuals times of the day were kept constant at pre and post testing. The tests were conducted mainly in the morning hours in a silent room while seated. The environment was quiet, without any noise or distraction. 

Concentration performance (CONP, sum of correctly crossed out characters minus overlooked symbols and falsely crossed characters) and error percentage (%ERR, sum of errors divided by amount of characters that have been processed) were included into further analyses. 

#### 2.2.3. Neuromuscular Outcomes

Static postural sway was assessed using a balance platform (GKS 1000^®^, IMM, Mittweida, Germany) [31]. This platform examines the center of pressure path length displacement (COP) by four strain gauges placed in the corners of the device. Each task was performed twice for 20 sec followed by a one minute break. Participants were instructed to stand on the platform as still as possible, to perform barefoot in a comfortable parallel feet position (shoulder width) and to keep the knees slightly bended with the hands placed on the hips while focusing on a marked spot on the wall (distance: 1.5 m; height: 1.75 m). 

Double limb stance with eyes closed (DLEC), single limb stance with eyes open (SLEO) without interference task and with cognitive and cognitive + motor interference tasks were tested. Counting backwards (subtracting three numbers starting from any number between 100 and 80) served as the cognitive interference task (CI). The motor inference task (MI) consisted of holding two interlocked sticks while trying to avoid contact between the two circles at the end of each stick. Single limb tasks were performed on the dominant leg. The dominant leg was determined using the lateral preference inventory [32]. Failed attempts (e.g., losing balance before 20 sec) were repeated. The best standing balance trial (shortest COP path length displacement) within the period of 5 to 15 sec was included into further analyses.

The heel rise test without additional loads (only body weight) was used to measure plantar flexor strength endurance [33]. We asked the participants to complete as many heel rises as possible on their dominant leg. Thereby, a moving frequency of 1 Hz was applied. This cadence was guaranteed by a metronome. Participants were instructed to perform without shoes. The knees were extended and the heels were not allowed to touch the ground. In order to maintain balance, it was recommended to touch the nearby wall with the index finger. Only heel rises exceeding the 50% mark of the range of motion (ROM) were taken into account. The total amount of correct heel rises served as outcome measure. Participants were asked to rate their subjectively perceived exertion immediately after exercise cessation. Therefore, the CR-10 BORG-scale was applied [34]. 

### 2.3. Point of Choice Prompt Intervention

All included participants were supplied with HAWDs from Office Plus (Ergon, 1.8 m × 0.72 m). A three-lined pop-up message (“prolonged sitting is harmful! (Attention)—Change your working position! (Affordance)—Lift up your working desk” (Action)) was used to promote standing time. This message appeared daily at 10:00, 13:00 and 15:00. Prompts appeared daily for a 12 week interventional period while the control group received no further information. However, the intervention group was not informed about the background of the appearance modalities prior to the start of the study. They only knew that three prompts were going to pop up within one workday.

These screen-based prompts were activated by personal user logins. Changing computers did not influence the appearance. Participants could immediately close their pop-up window after appearance by clicking the ok-button or x-field.

### 2.4. Statistical Analysis

Means and standard deviations (SD) were reported for all outcome measures. These outcomes were checked for variance homogeneity (Levene test) and normal distribution (Kolmogorov-Smirnov test). Data were analyzed using IBM SPSS Statistics 21. A significance level of *p* < 0.05 was set. All outcomes were analyzed via separate 2 (group: INT *vs*. CON) × 2 respectively 3 (time: pre, (mid), post) repeated measures analyses of variance (rANOVA). To assess the overall effect sizes for rANOVA, partial eta squared (η_p_²) was computed with η_p_² ≥ 0.01 indicating small, ≥0.059 medium and ≥0.138 large effects for each parameter [35]. Effect sizes of pairwise comparisons were reported by Cohen’s d (trivial: *d* < 0.2, small: 0.2 ≤ d < 0.5, moderate: 0.5 ≤ d < 0.8, large d ≥ 0.8). We also conducted analyses of covariance (ANCOVA) in order to adjust for potential baseline differences in the respective outcome variable. 

## 3. Results 

### 3.1. Sitting and Standing Time Proportions

**Figure 2:**
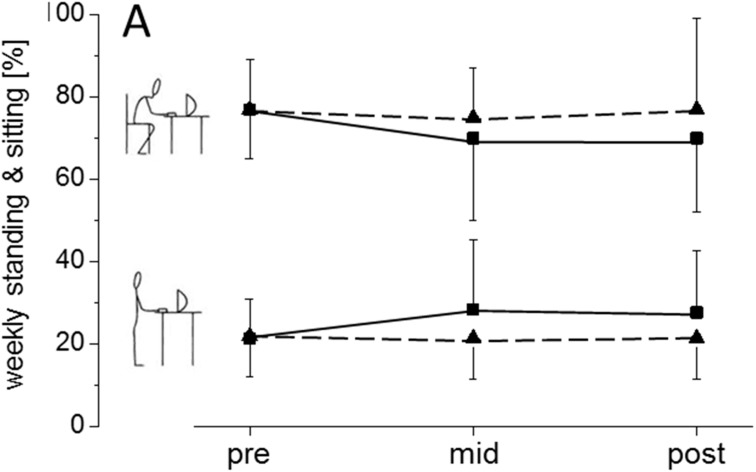
Percentage of occupational sitting and standing time at pre, after 6 weeks (mid) and 12 weeks (post) for INT (squares with solid line) and CON (triangles with dashed line). Data are depicted as means and standard deviations (SD). Pairwise effect sizes are provided as standardized mean differences (Cohen’s d).

We did not find significant group × time interactions for the percentage values of weekly sitting (*p* = 0.21, η_p_² = 0.05) and standing (*p* = 0.22, η_p_² = 0.05). Also no time effects were found for standing (*p* = 0.11, η_p_² = 0.07) and sitting (*p* = 0.07, η_p_² = 0.08) as well. The respective effect sizes for the main effects remained small and not significant. However, the pairwise effect size comparison revealed at least moderate to large between-group effects after 6 (mid) and 12 weeks (post), respectively (Figure 2). When comparing changes of standing time in hours, we found a moderate effect sizes of the group × time interaction (η_p_² = 0.09) with a trend towards statistical significance level (*p* = 0.09). *Post-hoc* testing revealed differences in sitting between pre and post testing for the INT (*p* = 0.03) (Table 2). The group time interactions slightly altered after adjusting for baseline differences (*p* = 0.07, η_p_² = 0.10). 

**Table 2:** Standing times (hours per week), concentration (d2-test), balance (postural sway) and strength (heel-rise test) performance for INT and CON during pre and post testings.

Performance Data	INT		CON		rANOVA			
	Pre	Post	Pre	Post	Time-Effect	η_p_^2^	Group × Time Interaction	η_p_^2^
Standing time (hours per week)	7.2 (4.8)	9.7 (6.6)	6.2 (3.0)	6.0 (3.0)	*p* = 0.18	0.06	*p* = 0.09	0.09
Sitting time (hours per week)	29.4 (6.5)	27.8 (10.7)	27.7 (9.5)	27.5 (9.2)	*p* = 0.55	0.01	*p* = 0.63	0.008
CONP	157 (52)	181 (52)	154 (36)	179 (37)	*p* < 0.001	0.73	*p* = 0.80	0.002
%ERR	6.0 (5.1)	4.1 (3.4)	9.8 (8.4)	6.0 (6.1)	*p* = 0.004	0.25	*p* = 0.52	0.01
DLEC	143 (27)	134 (17)	150 (34)	143 (25)	*p* = 0.09	0.09	*p* = 0.95	<0.001
SLEO	439 (129)	400 (126)	447 (158)	403 (143)	*p* = 0.009	0.21	*p* = 0.87	<0.001
SLEO + COG	514 (166)	449 (146)	492 (161)	473 (132)	*p* = 0.03	0.15	*p* = 0.23	0.05
SLEO + COG and +MOT	472 (175)	466 (154)	512 (163)	481 (158)	*p* = 0.20	0.06	*p* = 0.38	0.02
repetitions	37 (16)	34 (18)	35 (14)	34 (13)	*p* = 0.44	0.02	*p* = 0.76	0.003
perceived exertion	7 (2)	7 (2)	7 (2)	7 (2)	*p* = 0.92	<0.001	*p* = 0.70	0.005

Concentration outcome (CONP); percentage errors (%ERR); double limb stance eyes closed (DLEC), single limb stance eyes open (SLEO); plus cognitive interference task (+COG); plus COG and motor interference task (+MOT). Data are shown as means with standard deviations (SD). Effect sizes are given as partial eta squared (ηp2). Significance level was set at *p* < 0.001 ***, *p* < 0.01 ** and *p* < 0.05 *. Data are given as *p*-values calculated from the repeated measure analyses of variance (rANOVA) and as mean ± SD.

### 3.2. Concentration 

No time × group interaction effects were found for concentration performance (CONP) and percentage of occurred errors (%ERR) (Table 2). Due to familiarization, very large time effects have been observed for both concentration parameters. ANCOVA analyses did not change these results.

### 3.3. Strength-Endurance and Balance Outcome

Neither time × group interaction effects were found for strength-endurance including perceived exertion level (0.70 < *p* < 0.76, 0.003 < η_p_² < 0.005) nor balance performance under all conditions (0.23 < *p* < 0.95, 0.001 < η_p_² < 0.05) (Table 2). Merely moderate to large time-effects have been observed for standing balance performance excluding the cognitive plus motor interference task (0.009 < *p* < 0.03, 0.09 < η_p_² < 0.21). ANCOVA analyses did not change after adjusting for baseline differences.

## 4. Discussion 

Although some studies investigated the use of point of choice prompts and the applicability of sit and stand desks, this is the first study that evaluated the application of daily low-frequently appearing screen-based prompts on changes from occupational sitting to standing in office workers. Our study revealed that prompts appearing three times a day resulted in notable changes (with moderate to large pairwise between group effects) of relative (%) and absolute (h/week) occupational sitting and standing time, respectively. Neuromuscular (standing balance under single and multitask and strength endurance) and concentration outcomes did not change after this intervention. 

Half of INT (n = 7) achieved more than 60 min of daily occupational standing time. None of the control group participants achieved such changes. The energy expenditure of 60 min standing has the potential to result in beneficial changes of relevant cardiovascular health outcomes [36]. However, this group is apparently small and we did not measure these parameters directly. The observation that people respond differently to the appearance of point of choice prompts is in line with previous studies on individual patterns of how prompts appeal to the participants [37,38]. These studies pointed to personal (e.g., socio-economical, volitional, motivation, educational) and environmental factors (job profile, applicability, reasoning of the prompt target) that can affect the compliance. In this regard, stressful jobs [39] and the presence of health impairments [40] are negatively associated with the use of HAWDs. Thus, it seems reasonable to assume that the application of point of choice prompts [41] should reflect individuals’ needs, backgrounds and the specific working environments [42,43] that, as a consequence, might enable a more successful occupational health promotion in terms of sit-to stand transfers. Future research should focus on inter-individual differences and environmental factors that can affect compliance and efficacy of point of choice prompt interventions. As working in standing position could be regarded as uncomfortable and demanding, a longer and progressive adaption phase can minimize the risk of falling back into previous comfortable behavioral patterns, such as prolonged sitting [43].

Although the employees were supplied with HAWDs prior to the start of the intervention period, these desks were not used previously. As a consequence, the baseline values for weekly occupational sitting time in the present study (approximately 6 h daily, 65% to 80%) are rather comparable with those of other studies [7,10,13,44,45]. INT revealed meaningful absolute and relative changes of daily standing time. According to the meta-analysis of Chau and coworkers [3], however, such rather small but meaningful increases of occupational standing time ranging from 30 to 60 min per workday could gain relevant long-term impacts on health outcomes from an epidemiological viewpoint. It has been stated that comparatively large office time reductions around 90 min per workday can only be achieved by employing complex and costly multi-component programs including consulting, education, scheduling, coaching and prompting [16]. Long-term effects of such interventional approaches regarding costs, impact and return on investments remain still unclear; particularly since time-consuming multicomponent approaches could be bothering with a perceived loss in productivity from an employee and employer viewpoint [46]. Consequently, the respective organizations should pay attention on keeping such strategies to an effective minimum [19]. Health promoting worksite programs should be embedded in policy strategies that aim at tackling unhealthy behavior in general. 

As already low-intensity exercise has been reported to reveal beneficial impact on concentration [47], the influence of standing time on concentration outcome is still controversially discussed and not completely understood yet. Whereas interrupted prolonged sitting can positively influence the work-focus and -attention due to increased cerebral perfusion [12], unfamiliar and uncomfortable standing position during work could also attenuate these effects resulting in decreased work performance, particularly in overweight persons [48]. However, an alternating switch between sitting and standing at work can contribute to an improved postprandial glucose response and, thus, might diminish overweight-related metabolic dysfunction [49]. This effect might improve health profiles with less work absences in the long-term. However, such studies to disentangle these aspects are still needed in the future. 

The small changes of standing time in the present study did not yield adequate stimuli to improve balance performance and strength-endurance. Although upright standing has been considered an appropriate static balance stimulus [50], double limb standing at the office do not deliver meaningful neuromuscular adaptations, especially in comparatively healthy young participants. This assumption seems to also account for strength improvements. In order to improve strength measures (maximal and explosive strength) more specific sensorimotor work-site training regimes are required. These regimes could be delivered in several bouts of balance training spread over the working day [22]. Sensorimotor balance training can yield transfer effects to force development (explosive strength) by altering spinal reflex activity (withdrawal of presynaptic inhibition of Ia pathways) [51]. Thus, the integration of sensorimotor training into worksite exercise training studies in general [52] and sit to stand approaches in particular might induce meaning improvements of neuromuscular performance in occupational environments.

Some study limitations need to be mentioned. The results should be interpreted with caution due to its limited representativeness. A small study population and moderate dropout rate additionally lower the study power. The participants were recruited from one company. Cross talk effects between employees of INT and CON cannot completely ruled out. At baseline measurement, the participants reported some difficulties when wearing the ActiGraph (tight pressure of the elastic fixation belt). Instead of the commonly used ActivPal we used the ActiGraph. The ActiGraph has been reported to serve as a valid instrument to distinguish between standing and sitting posture [27]. The application on the thigh delivers some inaccuracy concerning the assessment of general physical activity [27]. The comparativeness of our results also at baseline with other studies is limited. Taking the variability of reported levels of sitting time in office workers, along with the differences in measurement method, and work environment (e.g., country, type of organization, employees, work performed) into account, we cannot address how much the employees with HAWDs in this study sat or stood before they got their desks. For example, Ryan *et al.* [10] and Evans *et al.* [7] used similar measurements (activPAL) in the same country and organization for similar types of workers (employed by the same university), but the group difference in mean percentage sitting time at work was about 10% (66% *vs.* 77%). The number of sit-stand-transfer and duration of the periods were not assessed. This information allows us to propose an interrelation between the length of standing periods and overall reductions of sitting time.

## 5. Conclusions 

In conclusion, the applied prompt intervention served as a cost-saving instrument and is easy to administer. The observations in a real life office setting and the undisturbed daily work routine during the study were further strengths. Further research is needed to investigate the effects of varying frequency patterns and delay-options of computer-based prompts including more elements of neuromuscular training in the occupational context.
